# Late-differentiated effector neoantigen-specific CD8+ T cells are enriched in peripheral blood of non-small cell lung carcinoma patients responding to atezolizumab treatment

**DOI:** 10.1186/s40425-019-0695-9

**Published:** 2019-09-12

**Authors:** Michael Fehlings, Suchit Jhunjhunwala, Marcin Kowanetz, William E. O’Gorman, Priti S. Hegde, Hermi Sumatoh, Boon Heng Lee, Alessandra Nardin, Etienne Becht, Susan Flynn, Marcus Ballinger, Evan W. Newell, Mahesh Yadav

**Affiliations:** 1 Genentech, 1 DNA way, South San Francisco, CA 94080 USA; 2 immunoSCAPE Pte Ltd, Singapore, Singapore; 3 Agency for Science, Technology and Research (A*STAR), Singapore Immunology Network (SIgN), Singapore, Singapore

**Keywords:** Immunotherapy, Atezolizumab, NSCLC, Tumor neoantigen-specific T cells

## Abstract

**Background:**

There is strong evidence that immunotherapy-mediated tumor rejection can be driven by tumor-specific CD8+ T cells reinvigorated to recognize neoantigens derived from tumor somatic mutations. Thus, the frequencies or characteristics of tumor-reactive, mutation-specific CD8+ T cells could be used as biomarkers of an anti-tumor response. However, such neoantigen-specific T cells are difficult to reliably identify due to their low frequency in peripheral blood and wide range of potential epitope specificities.

**Methods:**

Peripheral blood mononuclear cells (PBMC) from 14 non-small cell lung cancer (NSCLC) patients were collected pre- and post-treatment with the anti-PD-L1 antibody atezolizumab. Using whole exome sequencing and RNA sequencing we identified tumor neoantigens that are predicted to bind to major histocompatibility complex class I (MHC-I) and utilized mass cytometry, together with cellular ‘barcoding’, to profile immune cells from patients with objective response to therapy (*n* = 8) and those with progressive disease (*n* = 6). In parallel, a highly-multiplexed combinatorial tetramer staining was used to screen antigen-specific CD8+ T cells in peripheral blood for 782 candidate tumor neoantigens and 71 known viral-derived control peptide epitopes across all patient samples.

**Results:**

No significant treatment- or response associated phenotypic difference were measured in bulk CD8+ T cells. Multiplexed peptide-MHC multimer staining detected 20 different neoantigen-specific T cell populations, as well as T cells specific for viral control antigens. Not only were neoantigen-specific T cells more frequently detected in responding patients, their phenotypes were also almost entirely distinct. Neoantigen-specific T cells from responder patients typically showed a differentiated effector phenotype, most like Cytomegalovirus (CMV) and some types of Epstein-Barr virus (EBV)-specific CD8+ T cells. In contrast, more memory-like phenotypic profiles were observed for neoantigen-specific CD8+ T cells from patients with progressive disease.

**Conclusion:**

This study demonstrates that neoantigen-specific T cells can be detected in peripheral blood in non-small cell lung cancer (NSCLC) patients during anti-PD-L1 therapy. Patients with an objective response had an enrichment of neoantigen-reactive T cells and these cells showed a phenotype that differed from patients without a response. These findings suggest the ex vivo identification, characterization, and longitudinal follow-up of rare tumor-specific differentiated effector neoantigen-specific T cells may be useful in predicting response to checkpoint blockade.

**Trial registration:**

POPLAR trial NCT01903993.

**Electronic supplementary material:**

The online version of this article (10.1186/s40425-019-0695-9) contains supplementary material, which is available to authorized users.

## Background

Blockade of immune checkpoints such as PD-L1 or PD-1 can induce cancer regression through activation of T cell responses directed against the tumor. Clinical trials with PD-1 and PD-L1 inhibitors have demonstrated consistent therapeutic responses in patients with advanced melanoma and NSCLC and are currently being tested in many other cancer types. However, despite these encouraging results, typically only a fraction of patients show a durable response to therapy and most patients do not derive any benefit at all [1–4]. The lack of response upon anti-PD-1/L1 therapy has been attributed to the absence of pre-existing anti-tumor T cell response, which is thought to be a prerequisite for the checkpoint blockade-mediated restoration of anti-tumor T cell activity [5, 6]. The cellular mechanisms underlying activation of anti-tumor T cell responses through PD-1/L1 blockade are not completely clear. It has been hypothesized that blockade of PD-1 and PD-L1 reinvigorates neoantigen-specific T cells resulting in killing of tumor cells [7–10]. Neoantigens derived from tumor-specific mutations can be presented on the surface of tumor cells and could drive tumor-specific T cell responses. Indeed, high mutational burden has been correlated with clinical response to checkpoint blockade in multiple indications [10–13]. However, direct evidence to link induction of neoantigen-specific T cell responses to clinical benefit during checkpoint blockade is scarce [7, 10, 14]. In fact, anti-tumor T cell responses that form the basis of checkpoint blockade-mediated immune activation are not well-established. In tumor infiltrates, co-expression of CD103 and CD39 has been shown on tumor-reactive T cells in cancer patients, and the presence of these T cells has been linked to response to immunotherapy [15–17]. Several groups have also attempted to investigate anti-tumor T cell responses in peripheral blood by measuring quantitative and qualitative changes in peripheral CD8+ T cells during checkpoint blockade. For instance, expression of PD-1 has been shown to enrich for tumor-reactive cells derived from peripheral blood in some contexts [18, 19]. Others have observed that pharmacodynamic changes, such as increased Ki-67 expression in T cells in the peripheral blood, can be suggestive of an anti-tumor T cell response. Wherry and Ahmed groups showed that treatment with anti-PD-1 results in an increase in proliferation of CD8+ T cells in the periphery which, however, is not completely specific to patients responding to immunotherapy [8, 9].

Despite these reports, it has been challenging to quantitatively and qualitatively define the characteristics of an effective anti-tumor T cell response during immunotherapy. The presence of neoantigen-specific T cells could be one way to measure the quality of the T cell response. However, our understanding of neoantigen-specific T cells in human cancers is fairly limited. Though it has been extensively studied in preclinical mouse models, prevalence of neoantigen-specific T cell responses in human cancers is still poorly understood. Their detection in human cancers has mostly been limited to tumor tissues - an analysis that is difficult to universally implement owing to limited availability of patient tumor samples. In fact, the extent to which neoantigen-reactive T cells circulate in the peripheral blood of cancer patients and whether their phenotype and frequency change during immunotherapy remains unclear and further insight has been hampered due to the challenge of analyzing rare T cell populations potentially reactive for hundreds of putative antigen specificities [7, 10, 14]. The detection of circulating neoantigen-specific CD8+ T cells in cancer patients has been historically challenging due to many factors, including the rarity of these cells in the circulation (they are likely enriched within tumors but may or may not recirculate), and the limitations of neoepitope candidate prediction algorithms, which may result in identification of epitopes that are not presented by the tumor cells and not immunogenic [20, 21]. In this study, we investigated quantity and quality of CD8+ T cell responses associated with anti-PD-L1 antibody (atezolizumab) treatment in PBMCs from NSCLC patients using mass cytometry and highly-multiplexed combinatorial tetramer staining to longitudinally monitor neoantigen-specific CD8+ T cells in patients with partial response or progressive disease upon treatment.

## Methods

### Study design and patient samples

A total of 28 samples of frozen peripheral blood mononuclear cells (PBMC) from 14 patients with NSCLC treated with atezolizumab from the POPLAR trial NCT01903993 were used in this study [22] (POPLAR trial, Additional file 4: Table S1). POPLAR (NCT01903993) was a multicenter, open-label, randomized, phase 2 study of atezolizumab compared with docetaxel in patients with NSCLC after platinum chemotherapy failure [22]. The trial was sponsored by Genentech, Inc., a member of the Roche Group, which provided the study drug, atezolizumab. The protocols and their amendments were approved by the relevant institutional review boards or ethics committees, and all participants provided written informed consent. The clinical trial was conducted in accordance with the Declaration of Helsinki and International Conference on Harmonization Guidelines for Good Clinical Practice: ClinicalTrials.gov: NCT01903993 (https://clinicaltrials.gov/ct2/show/NCT01903993). All patients had measurable disease at baseline. RECIST v1.1 was used to assess response to therapy. Fourteen patients were randomly chosen based on the availability of PBMCs for analysis and clinical response, to yield roughly equal numbers of those who had an objective response, as assessed by RECIST v1.1, and those who progressed on atezolizumab therapy.

### Neoantigen prediction

Whole exome sequencing data was generated from tumors of 14 NSCLC patients and neoantigens were predicted as described previously [23]. Briefly, DNA for sequencing was extracted from both tumor and PBMCs using the Agilent SureSelect v5 (51 MB) kit on a HiSeq 2500 (Illumina®) sequencer.

Somatic variants were called using a union of Lofreq v2.1.2 [24] and Strelka calls [25]. Somatic mutations were annotated for effects on transcripts using Ensembl Variant Effect Predictor [26] on RefSeq-based gene models.

In order to identify expressed mutations, RNAseq alignments were tallied for somatic mutations identified in the exome data using the tallyVariants function from the R package VariantTools (v1.12.0; Bioconductor). Mutations with 2 or more RNA reads concordant with the mutation were retained, while other mutations that did not have any evidence in RNA-seq data were discarded. HLA genotyping was done on whole exome data from PBMCs, using Polysolver [27]. For each somatic mutation resulting in an amino acid change in a protein, all 8-11mer peptides from the protein that contain the mutation were considered as candidate neoepitopes. The binding affinity of each of these candidate neoepitopes to each HLA allele from the subject was predicted using NetMHCcons-1.1. We chose to use NetMHCcons for prediction because at the time of analysis of this study, NetMHCcons performed among the best prediction algorithms, as benchmarked by IEDB on a weekly basis. Other methods like IEDB_consensus performed comparably but not better than NetMHCcons. Neoantigen potential of each mutation was predicted after identifying HLA genotypes of the subjects, and assigning the optimal HLA-neoepitope pair across all HLA alleles and 8-11mer peptides containing the mutation, based on minimum IC50 values predicted by NetMHCcons [28].

### Peptide selection for tetramer generation

For tetramer generation we selected peptides predicted to bind to the alleles expressed by selected patients that could be tested in our system (“HLA-A*02:01”, “HLA-A*01:01”, “HLA-A*03:01”, “HLA-A*11:01”, “HLA-A*24:02”, “HLA-B*07:02”). An IC50 cutoff of 500 nM was used to identify all candidate neoepitopes that may bind to these alleles. Thus, the optimal predicted neoepitopes may not necessarily be included in this set of peptides, due to the specific set of alleles considered. All peptides resulting from the predicted binding affinity IC50 value of less than 500 nm were selected for the generation of tetramers (Additional file 5: Table S2).

### Tetramer generation

A total of 782 neoantigen peptides, synthetized by Mimotopes, Australia, with a purity above 85%, were used for tetramer construction (Additional file 5: Table S2). For each of the HLA alleles in the present study, up to 19 different control peptides (virus or patient tumor-unrelated epitopes) were also tested (Additional file 5: Table S2).

To screen for neoantigen-specific CD8+ T cells, a three-metal combinatorial tetramer staining approach was used, as described previously [29, 30]. This approach allowed us to simultaneously analyze hundreds of multiple candidate neoantigen peptides in a single patient sample using limited amount PBMCs without the need to stimulate or culture the cells (outlined in Additional file 1: Figure S1). Briefly, specific peptide-MHC class I complexes were generated by incubating UV-cleavable peptide–MHC class I complexes in the presence of individual candidate antigens. For the generation of a triple-coded tetramer staining mixture, three out of 12 differently heavy metal-labelled streptavidins were randomly combined resulting in a total of 220 unique barcode combinations. For internal validations we set-up a second configuration staining using a completely different barcoding scheme [30]. For tetramerization, these mixtures were incubated with the exchanged peptide–MHC complexes at a final molar ratio of 1:4 (total streptavidin:peptide–MHC). The tetramerized peptide–MHC complexes were combined, concentrated (10 kDa cutoff filter) and exchanged into cytometry buffer (PBS, 2% fetal calf serum, 2 mM EDTA, 0.05% sodium azide) before staining the cells.

### Phenotypic panel set-up

Purified antibodies lacking carrier proteins (100 μg/antibody) were conjugated to MAXPAR® DN3 metal chelating polymers loaded with heavy metal isotopes according to the manufacturer’s recommendations (Fluidigm). A specific antibody staining panel was set up consisting of lineage markers (CD45, CD14, TCRγδ, CD3, CD4, CD8, CD56, CD16), descriptive markers (CD57, HLA-DR, CD49a, CD69, CD45RO, OX40, CD103, CD38, KLRG-1, ICOS, TIGIT, CD27, PD-1, Tim-3, CD127, CD161, CCR7, CD25, 2B4, CD28, CD39) (Additional file 6: Table S3), labels for live/dead discrimination (cisplatin) and DNA (iridium intercalator), as well as five channels for different palladium metals used for sample barcoding. All labelled antibodies were titrated and tested by assessing the relative marker expression intensity on relevant immune cell subsets in PBMC from healthy donors.

### Sample staining and acquisition

Samples were thawed at 37 °C and transferred into complete RPMI medium 10% hiFCS (fetal calf serum), 1% penicillin/streptomycin/glutamine, 10 mM HEPES, 55 μM 2-mercaptoethanol (2-ME) supplemented with 50 U/ml Benzonase (Sigma) and immediately processed for staining. Since considerable variation in sample quality was observed, a sorting step was implemented for some of the samples to overcome poor sample quality, which may result in higher background or cell loss during sample staining. Therefore, cells were stained with fluorescently-conjugated (allophycocyanin, APC) anti-human CD45 antibodies (BioLegend) and Live/Dead (ThermoFisher) cell stain on ice for 20 min. Subsequently cells were washed twice and live CD45-positive lymphocytes were sorted using an ARIA II flow cytometry cell sorting device (Beckton Dickinson). Sorted cells were then added to healthy donor PBMC to reach a minimum of 3 × 10^6^ cells per staining condition. To discriminate live from dead cells, each sample was incubated for 5 min on ice in 200 μM cisplatin. Cells were then washed and stained with 100 μl of tetramer cocktail for 1 h at room temperature (RT). For antibody staining, samples were stained with a primary fluorescently-labelled anti-TCRγδ antibody for 30 min on ice, washed twice, then incubated with 50 μl of metal-labelled antibody cocktail for 30 min on ice, followed by fixing in 2% paraformaldehyde in PBS overnight at 4 °C. Samples were then washed once in permeabilization buffer and barcoded with a unique combination of two distinct barcodes for 30 min on ice. Cells were washed once, incubated in cytometry buffer for 5 min, and then resuspended in 250 nM iridium intercalator (DNA staining) in 2% paraformaldehyde/PBS at RT. Cells were washed and samples from each patient were pooled together with 1% polystyrene bead standards (EQ™ Four element calibration beads, Fluidigm) for acquisition on a HELIOS mass cytometer (Fluidigm).

### Data and statistical analyses

Signals for each parameter were normalized based on equilibration beads (EQ™ Four Element Calibration Beads, Fluidigm) added to each sample [31]. Since mass cytometry provides absolute quantitation of isotopic metal labels bound to each cell, metal-conjugated antibodies that are not detected on single cells are measured as zero value. To improve visualization of cells displayed in a compressed 2-dimensional dot plot, we randomized the signal of zero into values between − 1 and 0 using R with flow Core package; this data processing does not affect further downstream analysis. Each sample was manually de-barcoded followed by gating on live CD8+ T cells (CD45+,DNA+,cisplatin-,CD3+ cells) after gating out Natural killer (NK) cells (CD56+,CD16+), monocytes (CD14+) and TCRγδ cells (CD3+,TCRγδ+) using FlowJo software (Tree Star Inc). APC-CD45-sorted patient samples could be distinguished from healthy donor PBMCs used for buffering through the inclusion of a heavy metal-labeled anti-APC antibody in the antibody staining cocktail (Additional file 6: Table S3). Patient samples were identified by gating on positive events in the anti-APC channel.

For the detection of triple-tetramer-positive cells we used an automated peptide-MHC gating strategy as previously described [30]. A cut off threshold (detection threshold based on total CD8+ T cell counts in each individual sample) was defined for the numbers of events to be detected in each staining configuration (≥2 for 2 configurations, ≥4 for 1 configuration staining). Events that did not pass the detection threshold were not taken into consideration for the subsequent criteria. For the analysis provided in the main figures, hits were considered when the frequencies of specific CD8+ T cell were greater than events from the CD4+ T cell gate or when we observed a high degree of correspondence between the two tetramer staining configurations (less than two-fold difference in ratio between the frequencies). To objectively assess the degree of confidence in calling each of these hits, we used additional metrics that are summarized below. The results of this analysis for each of the hits are summarized in Additional file 7: Table S4.

(i) Phenotypic homogeneity. To assess phenotype skewing of antigen-specific T cells we assessed phenotypic uniformness of target cells against a random set of unspecific bulk T cells through their position in the high dimensional space.
(ii) Frequency in patient samples versus healthy donor PBMC background (for neoantigens only). The frequencies of neoantigen-specific CD8+ T cell events in the patient sample were compared to the number of events in the corresponding gate in healthy donor PBMCs that were included in the same staining approach.

Phenotypic profiles were displayed using t-Distributed Stochastic Neighbor Embedding (t-SNE) for high-dimensionality reduction and heat-maps. For t-SNE, the cell events of all samples were down-sampled to a maximum number of 20,000 CD8+ T cells per sample. t-SNE analysis was carried out by using an R script that uses the “flowCore” and “Rtsne” CRAN R packages for an efficient implementation of t- SNE via the Barnes-Hut approximations as previously described [29]. In R, all data were transformed using the “logicleTransform” function by using the “flowCore” package (parameters: w = 0.25, t = 16,409, m = 4.5, a = 0). Bar graphs were generated using Graphpad Prism software and heat maps were generated using custom R-scripts. Dot plots and t-SNE plots were displayed using Flowjo.

## Results

### No significant treatment- or response-associated differences in profile of bulk CD8+ T cells in NSCLC patients treated with atezolizumab

To investigate the effects of PD-L1 blockade on overall T cell responses during cancer immunotherapy, we performed a mass cytometry-based analysis of CD8+ T cells derived from PBMCs from a cohort of 14 NSCLC patients treated with atezolizumab. Of these patients, eight and six were objectively classified as responders and non-responders, respectively [22]. PBMC samples from individual patients taken at baseline and/or during treatment were thawed, stained and barcoded together using a panel of up to 29 markers dedicated to T cell identification and profiling, including several markers of activation and co-stimulation, as well as inhibitory molecules and markers associated with T cell dysfunction. After acquisition, samples were de-barcoded into individual patient time points and gated on live CD8+ T cells followed by downstream analysis of marker expression profiles.

**Fig. 1:**
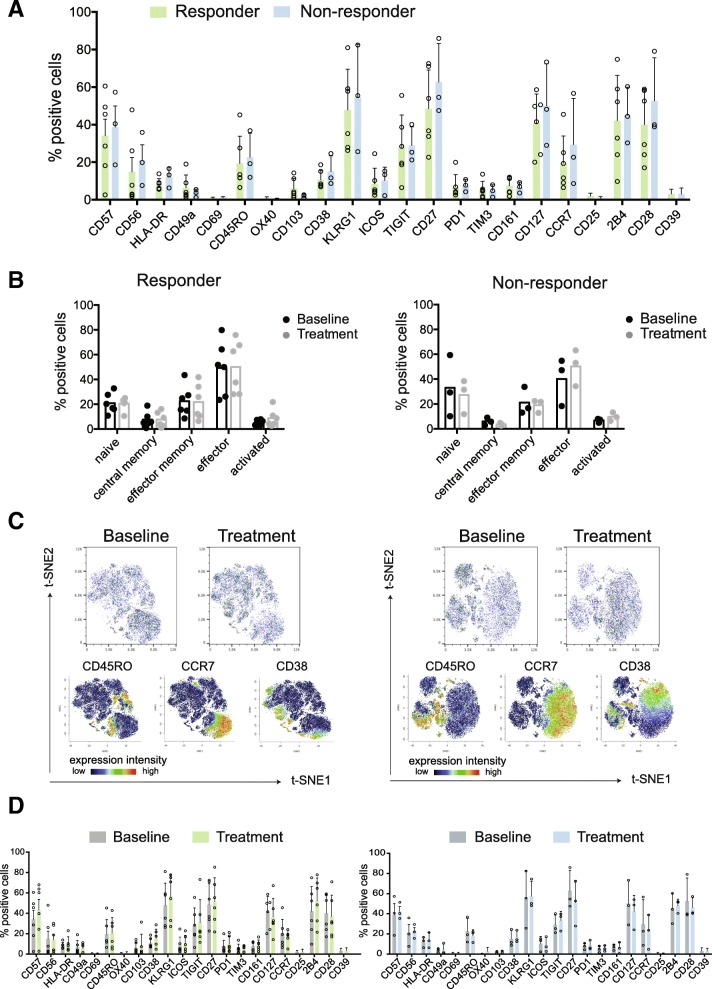
No difference in bulk CD8+ T cells phenotype at baseline or following treatment between atezolizumab responders and non-responders. **a** Frequencies of CD8+ T cells positive for all assessed marker molecules at baseline. **b** Frequencies of major CD8+ T cell subsets (naïve: CD45RO-,CCR7+; central memory: CD45RO+,CCR7+; effector memory: CD45RO+,CCR7-; effector: CD45RO-,CCR7-; and activated cells: CD38+/CCR7-) at baseline and on atezolizumab treatment. Each dot represents a patient. **c** Representative t-SNE map visualizing CD8+ T cells from one responder and one non-responder at baseline and on atezolizumab treatment with related plots showing relative position of cells expressing CD45RO, CCR7 and CD38. **d** Frequencies of CD8+ T cells positive for all analyzed markers at baseline and on atezolizumab treatment. Data shown from responders (green, *n* = 6) and non-responders (blue, *n* = 3)

For those patients with samples taken both pre- and on-treatment with atezolizumab (six responders and three non-responders), we assessed whether pre-treatment phenotypic profiles of CD8+ T cells differed between responding and non-responding patients. As summarized in Fig. 1a, frequency of most of the markers on CD8+ T cells did not significantly differ between the two groups.

We next separated T cells into subpopulations based on stages of T cell differentiation and activation and compared their frequencies at baseline and post atezolizumab treatment in responder and non-responder groups. Comparison of naïve (CD45RO-,CCR7+), central memory (CD45RO+,CCR7+), effector memory (CD45RO+,CCR7-), effector (CD45RO-,CCR7-), and activated cells (CD38+,CCR7-) from both groups did not show any significant differences (Fig. 1b), which could however be due to the relatively small numbers of samples.

We next applied the t-SNE algorithm [32, 33] for a high-dimensional visualization of the total phenotypes from the two groups before and after therapy initiation (Fig. 1c, Additional file 2: Figure S2). Consistent with previous findings [8, 9], we observed that CD8+ T cells, whether from responders or non-responders, are comprised of heterogeneous cell populations based on differential expression intensities for each phenotypic marker across the total CD8+ T cell population. Representative results from patients in the responder and non-responder groups are shown in Fig. 1c. To study potential treatment-associated changes within the responder and non-responder group, we compared frequencies of expression of all markers at both time points (baseline and on-treatment). In this dataset we did not detect significant differences in pre- versus on-treatment phenotypes of CD8+ T cells derived from individual patients treated with atezolizumab (Fig. 1d), and it is possible that the current dataset is too limited to reach statistical significance and that future studies will be needed to assess the robustness of these observations. Nonetheless, our results underline the challenges of using broad phenotypic profiling of bulk CD8+ T cells to identify correlates of clinical response at baseline or to assess biological activity of atezolizumab in NSCLC patients.

### Neoantigen-specific peripheral CD8+ T cells are enriched in NSCLC patients responding to treatment with atezolizumab

We investigated presence of neoantigen-specific CD8+ T cells in PBMC from responders and non-responders to better understand the effects of atezolizumab treatment on these cells. Accurate prediction of immunogenic neoepitopes has proven challenging, and typically only a fraction of predicted neoantigens are ultimately validated as truly immunogenic [14, 30, 34]. To sensitively detect neoantigen-specific CD8+ T cells ex vivo without an in vitro culture or stimulation bias, we used a mass cytometry-based combinatorial triple-coded multiplexed peptide-MHC tetramer staining approach, as previously described [29, 30, 35]. We conducted whole exome sequencing (WES) of DNA from tumor and matched normal blood samples from all 14 atezolizumab-treated patients. Tumor neoepitopes were predicted based on potential for MHC class I binding to patient-specific HLAs and selected for testing based on confirmed gene expression in the tumor (see Methods). This pipeline yielded MHC-tetramers for 782 peptides predicted to bind to 6 different patient HLA alleles: HLA-A*02:01, HLA-A*03:01, HLA-A*11:01, HLA-A*01:01, HLA-A*24:02 and HLA-B*07:02. In addition, we tested up to 19 different non-tumor control peptides per HLA for each sample (Additional file 5: Table S2). The average number of neoepitopes screened in responder and non-responder patient samples were 61 and 50, respectively, with a range of 1 to 139 neoepitopes per patient.

**Fig. 2:**
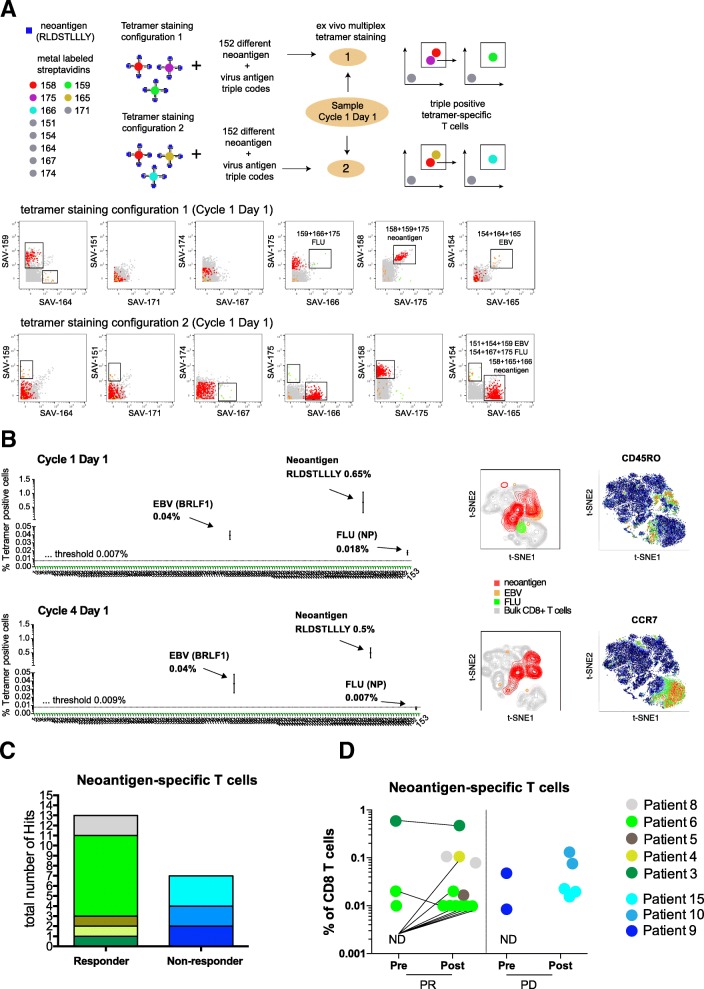
Neoantigen-specific T cells are enriched in patients responding to atezolizumab treatment. **a** Schematic overview of the multiplexed tetramer staining approach and corresponding example of identification of triple positive neoantigen and virus-specific T cells from a representative responder patient at baseline levels (cycle 1 day 1) in two staining configurations. Screening for antigen-specific CD8+ T cells was performed by using a mass cytometry-based multiplex triple coding tetramer staining approach assessing 153 candidate antigens, 126 neoantigens, and 30 cancer-unrelated control antigens for this patient. Each peptide-MHC was labelled with a unique combination of three heavy metal-streptavidin labels. **b** Same patient before (detection threshold 0.007%) and post atezolizumab treatment (detection threshold 0.009%). T cells specific for one neoantigen and two viral epitopes were identified based on the detection criteria set (see also Methods). t-SNE plots are based on the expression of all phenotypic markers. Relative expression levels of CCR7 and CD45RO are shown. **c** Total number of unique neoantigen-specific CD8+ T cells (hits) detected from a total of 782 neoantigen candidates within the responders (*n* = 8 patients) and non-responders (*n* = 6 patients) groups. **d** Frequencies of all neoantigen-specific CD8+ T cells detected within the responders (13 neoantigens) and non-responders (7 neoantigens) group pre- and post- atezolizumab treatment. The frequencies of T cells specific for neoantigens ranged from as low as 0.01% to as high as 0.65% of total CD8+ T cells. For patients where baseline sample was available but no antigen-specific T cells were detected are shown as N.D. Abbreviations: N.D., not detected; PR, responders; PD, non-responders

For the identification of antigen-specific T cells we used an automated combinatorial peptide-MHC gating strategy and defined objective criteria based on the detection limit, background noise, and consistency of technical replicates for a bona fide hit calling (see methods for details). Figure 2a shows an example of the identification of antigen-specific CD8+ T cells using two different tetramer staining configurations in NSCLC patient responding to atezolizumab therapy (Patient 3). PBMC samples from this patient obtained before and during treatment were screened for a total of 126 neoantigen candidates and 30 viral-specific nontumor control peptides. We detected a significant fraction of CD8+ T cells specific for an HLA-A*03:01-restricted neoantigen (RLDSTLLLY) present at initiation of the treatment (cycle 1, day 1) and also during treatment (cycle 4, day1), 0.65 and 0.5%, respectively). In addition, we detected T cells specific for one HLA-A*02:01-restricted EBV epitope at both cycles (BRFL-1, 0.039 and 0.037% of CD8+ T cells, respectively) and for one HLA-A*03:01-restricted influenza epitope at cycle 1 day 1 (NP, 0.018% of CD8+ T cells) in the same patient (Fig. 2b and see also Additional file 8: Table S5).

Amongst all 782 candidate tumor epitopes screened, we identified T cells reactive for 13 different neoantigens across all responders (five out of eight responders) and 7 neoantigen specificities across the non-responders (three out of six non-responders) (Fig. 2c, Additional file 9: Table S6). The frequency of neoantigen-specific CD8+ T cell ranged from as low as 0.01% to as high as 0.65% of CD8+ T cells (Fig. 2d) with a range of 1 to 8 neoepitope hits per patients. In most cases, we were able to reach a limit of detection of 0.02%; in some cases, T cells against neoepitopes could have been missed because of the higher limit of detection due to acquisition of fewer viable PBMC.

In summary, these data show a trend toward greater abundance of neoantigen-specific T cells in patients responding to atezolizumab treatment, with 13 out of 20 hits detected in responders. Also, neoantigen-specific T cell responses are further enriched post treatment in responders. Although validation in a larger study will be required, these findings do suggest that the presence of neoantigen-specific T cell responses at baseline or their expansion post-treatment could be associated with clinical response to checkpoint blockade.

### Neoantigen-specific CD8+ T cells in responder patients show a highly differentiated effector phenotype

**Fig. 3:**
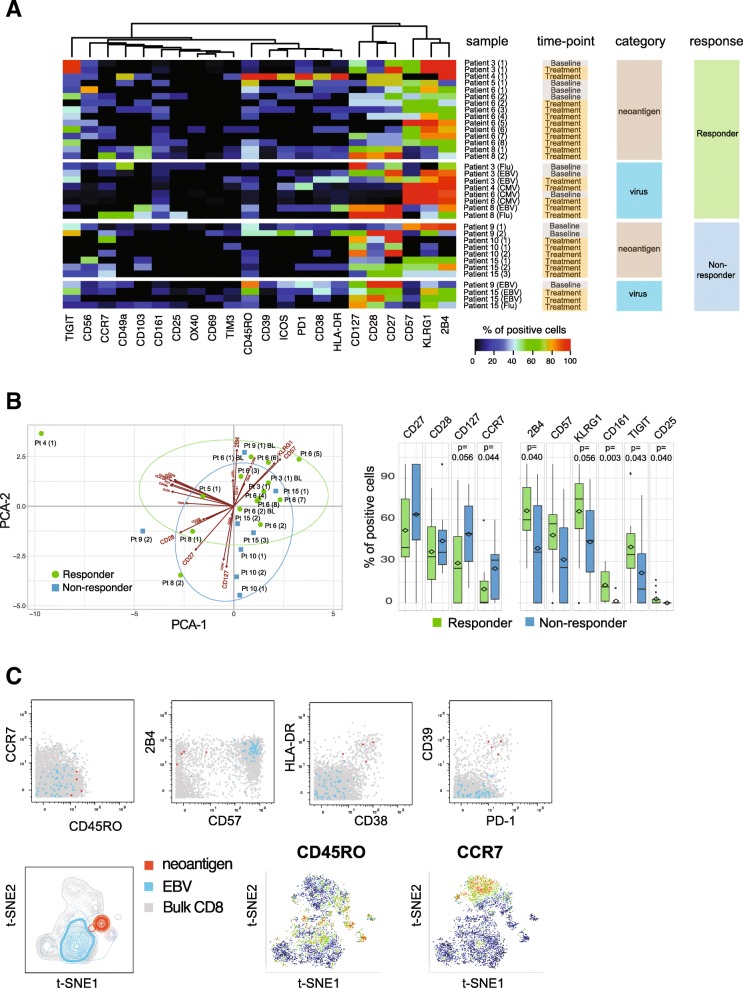
Neoantigen-specific T cells in atezolizumab responder patients show a more differentiated effector phenotype. **a** Heatmap representing frequency of antigen-specific CD8+ T cells positive for all phenotypic markers analyzed. Results for all neoantigen-specific and virus-specific CD8+ T cells detected in individual patients are shown, grouped by responders and non-responders. Markers are ordered based on unsupervised hierarchical clustering. Numbers in brackets correspond to unique neoantigens detected in each patient. **b** The first two components obtained from PCA of percentages of neoantigen-specific T cells for each marker are plotted for each hit (left). Boxplots show the trends toward a higher number of neoantigen-specific T cells positive for CD27, CD28, CD127, and CCR7 in the non-responder group and 2B4, KLRG-1, CD57, CD161, TIGIT, and CD25 in the responder group, respectively (Wilcoxon signed rank test). **c** Biaxial dot plots showing an example of neoantigen-specific T cells displaying an activated phenotype with co-expression of PD-1 and CD39. t-SNE plots are based on the expression of all phenotypic markers. Relative expression levels of CCR7 and CD45RO are shown. Data shown from Patient 4 (red, neoantigen-specific T cells; blue, EBV-specific T cells; grey, bulk CD8+ T cells)

Information on immune profiles of neoantigen-specific T cells is scarce as technical difficulties have impeded in-depth phenotyping of rare antigen-specific T cells in the past. Utilizing an antibody panel designed for CD8+ T cell profiling, we analyzed the phenotypes of all neoantigen-reactive T cells detected in either patient group in order to unravel potential qualitative differences in the nature of tumor-specific T cell responses. To objectively compare the phenotypes of antigen-specific T cells derived from different patients and time points, we determined the frequencies of cells expressing 22 distinct markers, using virus-specific T cells identified in these patients as benchmarks (Fig. 3a, Additional file 9: Table S6). Across patients, T cells specific for neoantigens displayed diverse phenotypic profiles with varying frequencies of cells expressing activation, co-stimulatory or inhibitory marker molecules. Interestingly, T cell phenotypes were mostly similar when pre- versus post-treatment samples from the same patient were compared (viz., Patients 3, 6, and 10, Fig. 3a and Additional file 3: Figure S3). However, among responders, CD8+ T cell phenotype was skewed toward higher expression of KLRG-1, 2B4, CD57, CD161, TIGIT, and CD25 than seen in non-responders, reflecting a late-differentiated effector phenotype. In contrast, the majority of antigen-specific T cells detected in non-responders showed a trend toward a higher expression of CD127, CD28, CD27, and CCR7 (Fig. 3b). Notably, amongst the responders we also detected neoantigen-specific T cells in one patient that were characterized by an activated phenotype (high HLA-DR and CD38 expression) as well as a high expression of PD-1 and CD39 (Fig. 3c). The expression of PD-1 and CD39 has recently been described in neoantigen-specific tumor-infiltrating lymphocytes (TILs) from colorectal cancer patients, and CD39 has been proposed as a marker for tumor-specific T cells [17, 29]. In addition, the expansion of CD39+ cells in the blood of patients receiving checkpoint blockade therapy has been reported [8], suggesting the CD39 expression we observed may be linked to recent treatment with atezolizumab.

**Fig. 4:**
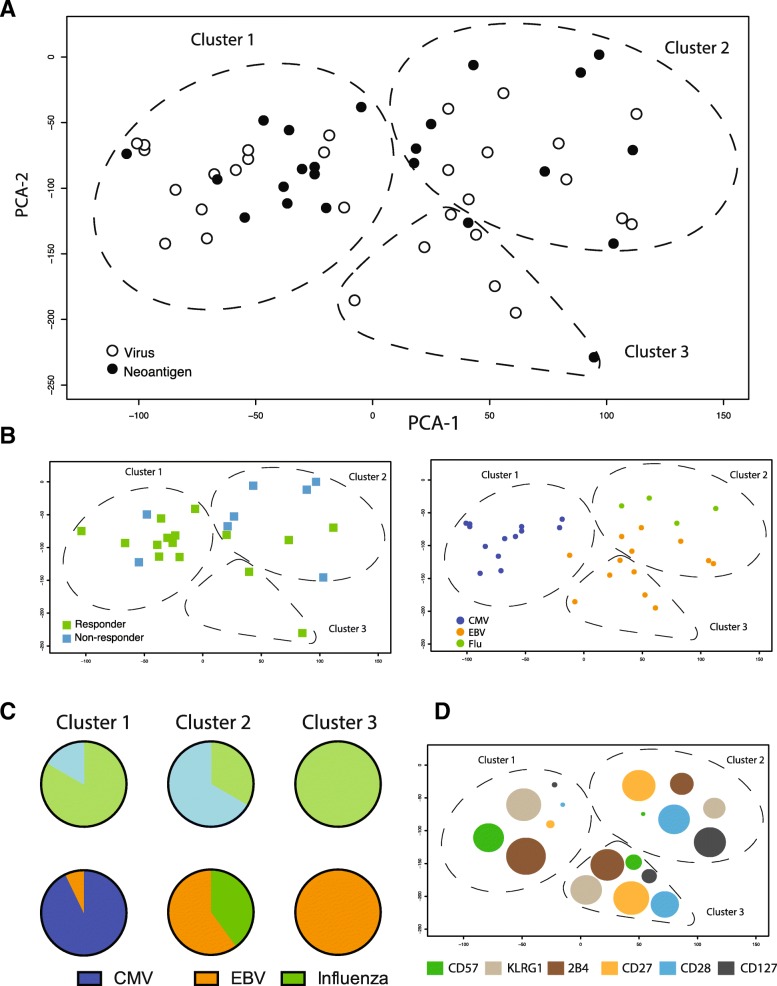
Neoantigen-specific T cells in atezolizumab responders are skewed towards a late differentiated CMV-like phenotype. **a** PCA of all neoantigen-and virus-specific CD8+ T cells hits identified in this study. PCA is based on phenotypic profiling (percent of antigen-specific CD8+ T cells positive for the markers shown in Fig. 3a). The distribution pattern of all hits across the first two principal components allows for an annotation of three distinct clusters. **b** The majority of the neoantigen-specific T cells from the responder group are located within Cluster 1 and 3, whereas most of the neoantigen-specific T cells from non-responder patients are detected in Cluster 2. CMV-specific T cells were mostly found in Cluster 1, EBV- and influenza-specific T cells mapped within Cluster 2 and 3. Labels are according to patient response and virus-specificity. **c** Pie chart summarizing the data shown in 4B: top, number of neoantigen hits; bottom, number of viral hits for each PCA cluster. **d** Graphical representation of the most differentially expressed markers of all virus-specific CD8+ T cells in the three PCA clusters; Bubble size is proportional to mean frequencies of all virus-specific CD8+ T cells positive for the indicated marker in any given cluster

In order to meaningfully compare the phenotypes of neoantigen-specific T cells with benchmark cancer-unrelated, virus-specific T cells derived from responder and non-responder patients, we reduced the high dimensionality of the dataset and plotted the phenotypic information from Fig. 3a as principal component analysis (PCA) showing the first two principal components in a two-dimensional graph. In this analysis, we also included data points from cancer-unrelated virus-specific T cells that were identified in all patients from the cohort (with or without detectable neoantigen specificities). The phenotypes of all antigen-specific T cells detected in this cohort could be segregated into three distinct arbitrary clusters with different degrees of overlap between the neoantigen and virus antigen-specific T cell populations (Fig. 4a). We found that 80% of the neoantigen-specific T cells from the responder group mapped within Cluster 1 and Cluster 3, while 75% of the neoantigen-specific T cells from non-responder patients were detected in Cluster 2 (Fig. 4b and c). Interestingly, Cluster 1 also consisted mainly of CMV-specific T cells, whereas Cluster 3 only included EBV-specific T cells. In contrast, a mixture of EBV- and influenza-specific T cells mapped within Cluster 2 (Fig. 4b and c).

The phenotypic segregation of neoantigen-specific T cells according to patients’ clinical response to atezolizumab treatment suggests these different functional characteristics could be critical to the response. Specifically, the late-differentiated CMV-specific T effector like phenotype (CD57-high, KLRG-1-high and 2B4-high, Fig. 4d) also seen in the majority of the neoantigen-specific T cells from atezolizumab responders may be associated with senescent cells with highly cytotoxic and strong anti-tumor activity [36]. In contrast, the neoantigen-specific T cells found within the non-responder group more often displayed a memory-like phenotype (CD27-high, CD28-high, and CD127-high) and may be less effective in carrying out an anti-tumor response.

## Discussion

This study was conducted to evaluate treatment-associated and response-associated changes in circulating neoantigen-specific T cells in NSCLC patients treated with atezolizumab. We used a multiplexing method for ex vivo identification and profiling of antigen-specific CD8+ T cells. We measured both quantitative (number of unique neoantigens hits, frequency of neoantigen-specific T cells) and qualitative (phenotype of neoantigen-specific T cells) properties of neoantigen-specific T cells and compared them phenotypically with CMV, EBV and influenza virus-specific CD8+ T cells found in the same group of patients.

Our findings bring new insight into the development of neoantigen-specific responses in cancer immunotherapy. In responder patients, we identified a heterogeneous population of neoantigen-specific CD8+ T cells with a late effector-like phenotype, which may be indicative of the functional state necessary to target antigens in the tumor. The observation that neoantigen-specific T cells are skewed towards specific functional phenotypes in patients with clinical response suggests that mere presence of endogenous tumor-reactive T cells may not be sufficient and that the quality of neoantigen-specific T cells could be a critical factor in predicting clinical outcome upon immunotherapy.

One of the advantages of our approach is to be able to screen for numerous antigen specificities with an extremely sensitive level of detection. This is critical because first only a minor fraction of tumor mutations are truly immunogenic [20, 37, 38] and second their frequencies are extremely low in peripheral blood [10, 12, 14]. In most previous studies, detection of neoantigen-specific cells was feasible only after cell expansion or re-stimulation [37, 38]; however, although functional assays are useful, ex vivo stimulation/culture is likely to lead to alteration of surface markers on the T cells and thus may not be able to inform on the true phenotype of T cells in vivo The ex vivo approach used here was sensitive enough to detect and characterize several neoantigen-specific T cell populations in patient PBMC, in most cases achieving a threshold of detection < 0.01% of CD8+ T cells. In our study, the patient-wide discovery rate was 20 unique hits out of 782 total neoantigen candidates tested, or 2.5%. This is within the range of previously reported studies analyzing ex vivo neoantigen-specific T cells, including those in TILs (for example, 0.5 to 2%, [39, 40]. The detection of some of these hits in multiple samples from the same patients, supports the reliability of the method and findings.

Although the number of neoantigen candidates predicted did not differ between responders and non-responders, the majority of CD8+ T cell responses against these neoantigens were found in patients with objective response to treatment. Overall, most of the phenotypic differences in neoantigen-specific cells observed in this study were reflective of patient clinical response to atezolizumab. Somewhat surprisingly, other than a few de novo hits detected post checkpoint inhibition in a couple of patients with partial response, we did not find many treatment-associated changes in those patients where a longitudinal follow-up was possible. Circulating frequencies of those neoantigen-specific T cells detected both before and after treatment were also similar, and we saw only minor changes in their phenotype, namely an increase in the activation markers CD57 and KLRG1 (see for example patient 3 and patient 6, Additional file 3: Figure S3). It is possible that the impact of treatment with respect to frequency and phenotype of tumor-reactive T cells is more prominent in the tumor microenvironment. In fact, pharmacodynamic changes in peripheral T cells during checkpoint blockade have not been clearly defined. Our previous study in preclinical tumor models aimed at characterizing the biological activity of checkpoint inhibitors showed that neoantigen-specific T cells in the tumor were reinvigorated and expanded upon treatment, where these specific T cells were previously most exhausted [29]. Observations from our current study may indicate that pre-existing, effector, tumor-reactive CD8+ T cells could be a prerequisite for anti-PD-L1 clinical efficacy, and blockade of PD-1/PD-L1 engagement may enable activation of CD8+ T cells in the tumor tissue, without radically perturbing their profile in the periphery.

The neoantigen-specific T cells in responder patients were universally characterized by relatively high expression of the activation markers CD161, TIGIT, 2B4 and KLRG1. CCR7 expression was also significantly lower than in non-responders. The expression of CD28 and CD27 has previously been shown to distinguish subsets of differentiated CD8+ T cells where downregulation of CD27 and CD28 was associated with late-differentiated CD8+ T cells [41]. Interestingly, some of the neoantigen-specific T cells from patients responding to atezolizumab in this study, also showed low level of expression of CD27, CD28 and CD127. Furthermore, these late-effector and effector-memory phenotypes have also been previously described in tumor-reactive TILs during T cell therapy [42]. Conversely, recent studies have also demonstrated that adoptively transferred transgenic T cells shift from a memory to a terminally differentiated effector phenotype over time [43]. These characteristics are indicative of functional, cytotoxic T cell populations like those capable of controlling persistent viral infections [41]. They imply recent antigen experience and suggest that an effective anti-tumor T cell response may be ongoing in these patients as suggested in previous studies in Hepatitis B virus infected patients [35]. Because of their limited proliferative abilities, however, differentiated effector T cells could be difficult to detect using assays and markers which rely on T cell expansion, and therefore might have been previously under-reported, yet critical for response to immunotherapy.

One limitation of our study is the small number of paired pre- and post-treatment patient samples. The small sample size prevented a statistically robust assessment of treatment and/or response-associated changes within circulating bulk CD8+ T cells and the limited availability of PBMC samples made it difficult to perform any additional functional assessment of the antigen-specific T cells. A direct comparison between nature and number of neoantigens-specific T cells in TILs and circulating PBMC in treated patients also remains of great interest but was not feasible in our settings.

## Conclusions

In conclusion, we have shown that neoantigen-specific T cells can be detected in peripheral blood in NSCLC patients during anti-PD-L1 therapy. Patients with an objective response had an enrichment of neoantigen-reactive T cells and these cells showed a phenotype that differed from patients without a response. Specifically, neoantigen-reactive T cells in patients with an objective response to atezolizumab have a differentiated effector phenotype, similar to that of CMV and/or EBV-specific CD8+ T cells. These observations imply that the unique phenotype of neoantigen-specific T cells and their resemblance to CMV-reactive T cells in responding patients may reflect the functional state of these T cells and their ability to attack tumor cells. Should further validation extend and confirm these findings, detection of effector, tumor-reactive T cells in the periphery could be developed to support patient selection for immune checkpoint inhibition strategies.

## Additional files

Additional file 1: **Figure S1.** Outline of methodology used for generating peptide-MHC tetramers and staining patient PBMCs. 1) Preparation of patient-specific peptide set and UV-cleavable peptide-MHC monomers. 2) UV-induced peptide exchange. 3) Preparation of randomly mixed triple streptavidin combinations. 4) Tetramerization of individual patient-specific peptide MHC monomers. 5) Combination of tetramerized peptide-MHC complexes and preparation of antibody mixture. 6) Sample thawing and direct staining with tetramers and antibody mixture for CyTOF analysis. (PDF 252 kb)

Additional file 2: **Figure S2.** High-dimensional immune profiles of CD8+ T cells from atezolizumab treated responders and non-responders. t-SNE maps display relative expression intensities of all phenotypic markers assessed. Shown are representative plots for CD8+ T cells and total CD45+ immune cells from one responder and one non-responder combined at baseline and on atezolizumab treatment. (PDF 25443 kb)

Additional file 3: **Figure S3.** Phenotypic profiles of neoantigen-specific T cells in patients pre- and post atezolizumab treatment. Heatmaps show median expression intensities of all phenotypic markers probed in samples derived from the same patients. Markers are ordered based on unsupervised hierarchical clustering. (PDF 510 kb)

Additional file 4: **Table S1.** Patient characteristics and list of PBMC samples selected for the current analysis from the POPLAR trial. (XLSX 11 kb)

Additional file 5: **Table S2.** Tab 1, Number of neoantigen and viral specific tetramers generated for each patient sample. Tab 2, Complete list of peptides used to generate tetramers with their corresponding HLA alleles and predicted binding affinity. (XLSX 41 kb)

Additional file 6: **Table S3.** List of antibodies, their clone information and heavy metal tags used in the staining panel for CyTOF. (XLSX 12 kb)

Additional file 7: **Table S4.** Complete list of tetramer hits for CD8+ T cells and information on additional metrics that were monitored for each hit. (XLSX 11 kb)

Additional file 8: **Table S5.** Neoantigen and virus epitope hits detected for patient 3. (XLSX 10 kb)

Additional file 9: **Table S6.** Complete list of all tetramer positive hits detected for neoantigens and viral epitopes for all patients in the current study. (XLSX 12 kb)

## Data Availability

The datasets supporting the conclusions of this article are included within the article and its additional files.
