# Supplementary material for: Late-differentiated effector neoantigen-specific CD8+ T cells are enriched in peripheral blood of non-small cell lung carcinoma patients responding to atezolizumab treatment
Source: J Immunother Cancer. 2019 Sep 12;7:249. doi: 10.1186/s40425-019-0695-9 (PMC6740011; doi:10.1186/s40425-019-0695-9)
Supplement: Supplementary file 1 — Figure S1. Outline of methodology used for generating peptide-MHC tetramers and staining patient PBMCs. 1) Preparation of patient-specific peptide set and UV-cleavable peptide-MHC monomers. 2) UV-induced peptide exchange. 3) Preparation of randomly mixed triple streptavidin combinations. 4) Tetramerization of individual patient-specific peptide MHC monomers. 5) Combination of tetramerized peptide-MHC complexes and preparation of antibody mixture. 6) Sample thawing and direct staining with tetramers and antibody mixture for CyTOF analysis. (PDF 252 kb) [file 40425_2019_695_MOESM1_ESM.pdf]

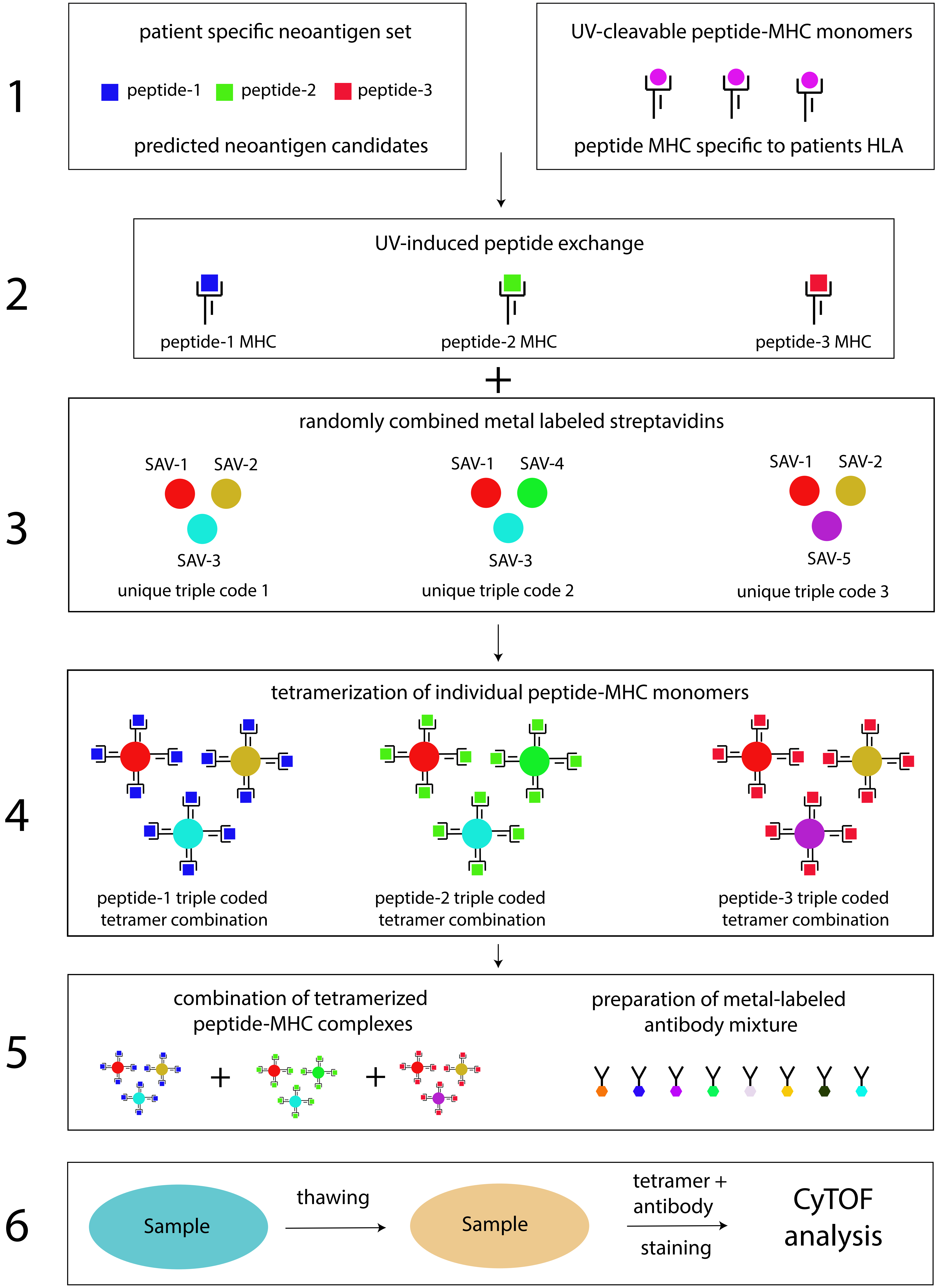
1
patient specific neoantigen set
peptide-1
peptide-2
peptide-3
predicted neoantigen candidates
UV-cleavable peptide-MHC monomers
peptide MHC specific to patients HLA
2
UV-induced peptide exchange
peptide-1 MHC
peptide-2 MHC
peptide-3 MHC
+
3
randomly combined metal labeled streptavidins
SAV-1
SAV-2
SAV-3
unique triple code 1
SAV-1
SAV-4
SAV-3
unique triple code 2
SAV-1
SAV-2
SAV-5
unique triple code 3
4
tetramerization of individual peptide-MHC monomers
peptide-1 triple coded tetramer combination
peptide-2 triple coded tetramer combination
peptide-3 triple coded tetramer combination
5
combination of tetramerized peptide-MHC complexes
preparation of metal-labeled antibody mixture
6
Sample
thawing
Sample
tetramer + antibody
staining
CyTOF analysis
