# Supplementary figures and images for: Late-differentiated effector neoantigen-specific CD8+ T cells are enriched in peripheral blood of non-small cell lung carcinoma patients responding to atezolizumab treatment
Source: J Immunother Cancer. 2019 Sep 12;7:249. doi: 10.1186/s40425-019-0695-9 (PMC6740011; doi:10.1186/s40425-019-0695-9)

Responder

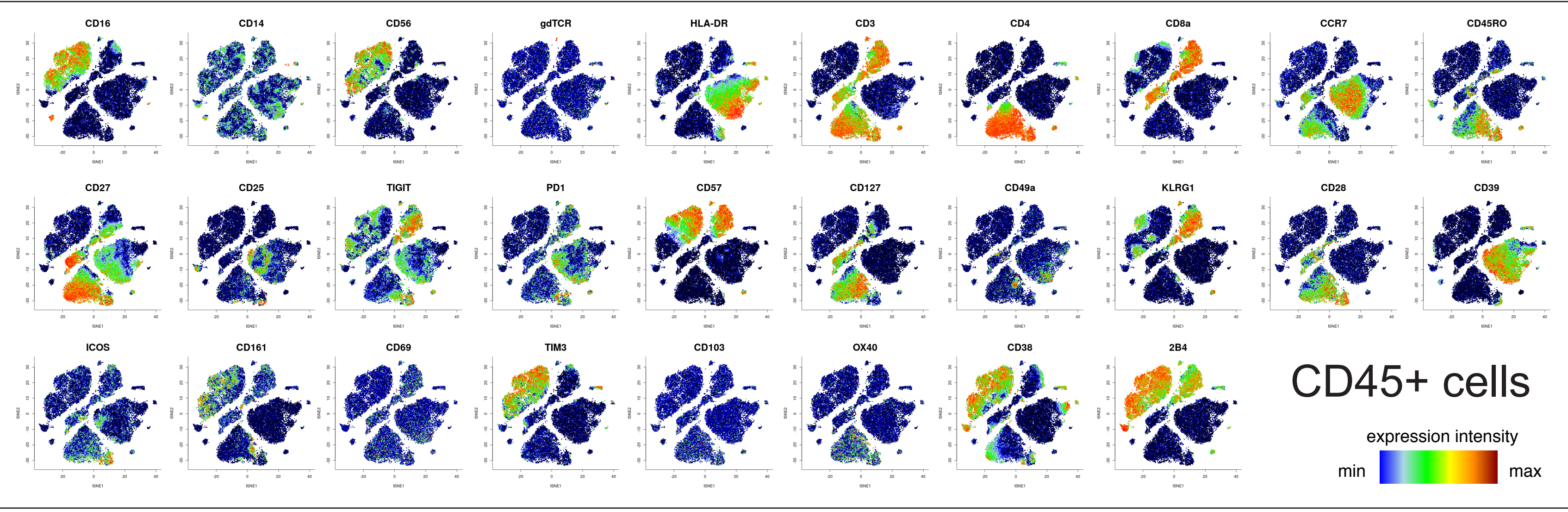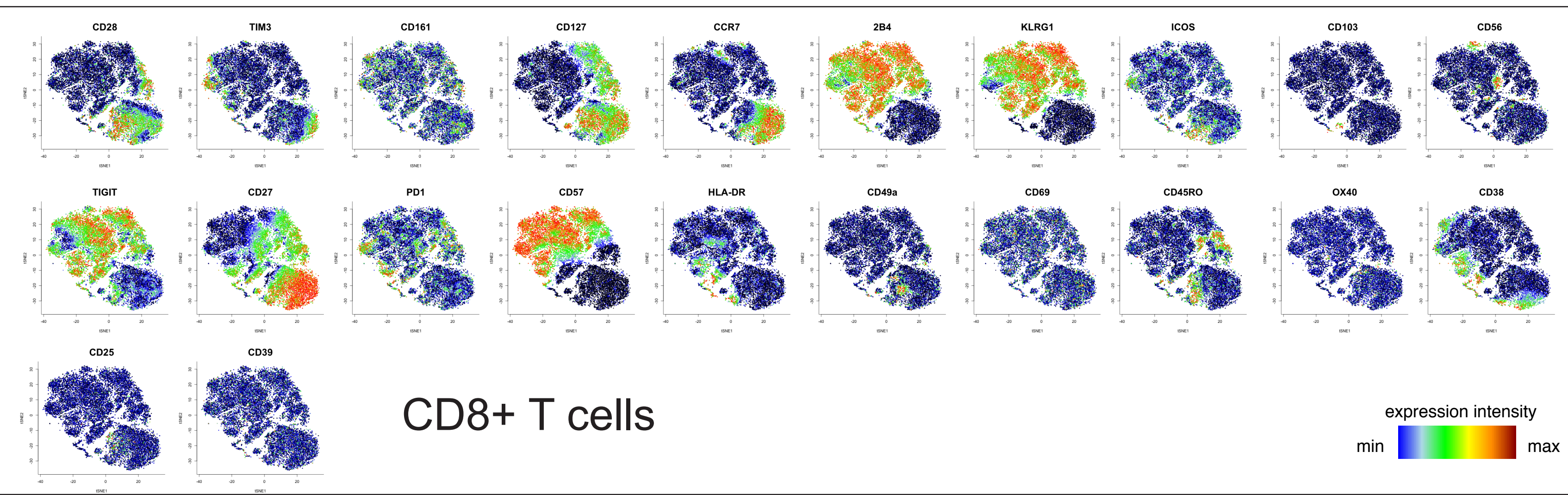

Non-responder

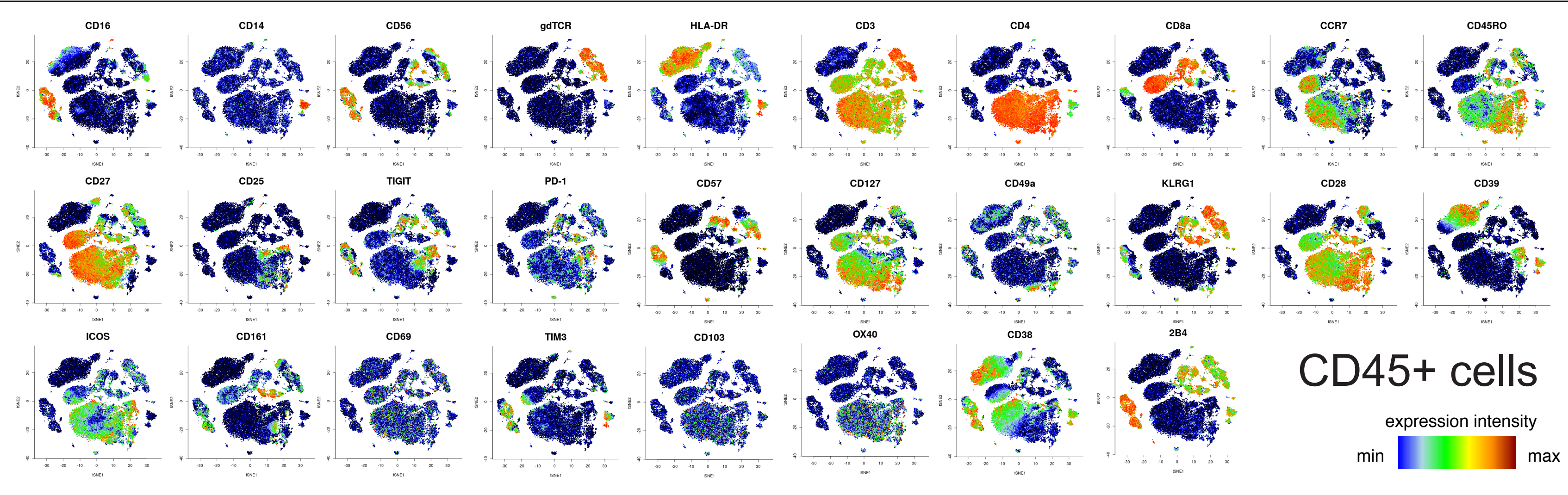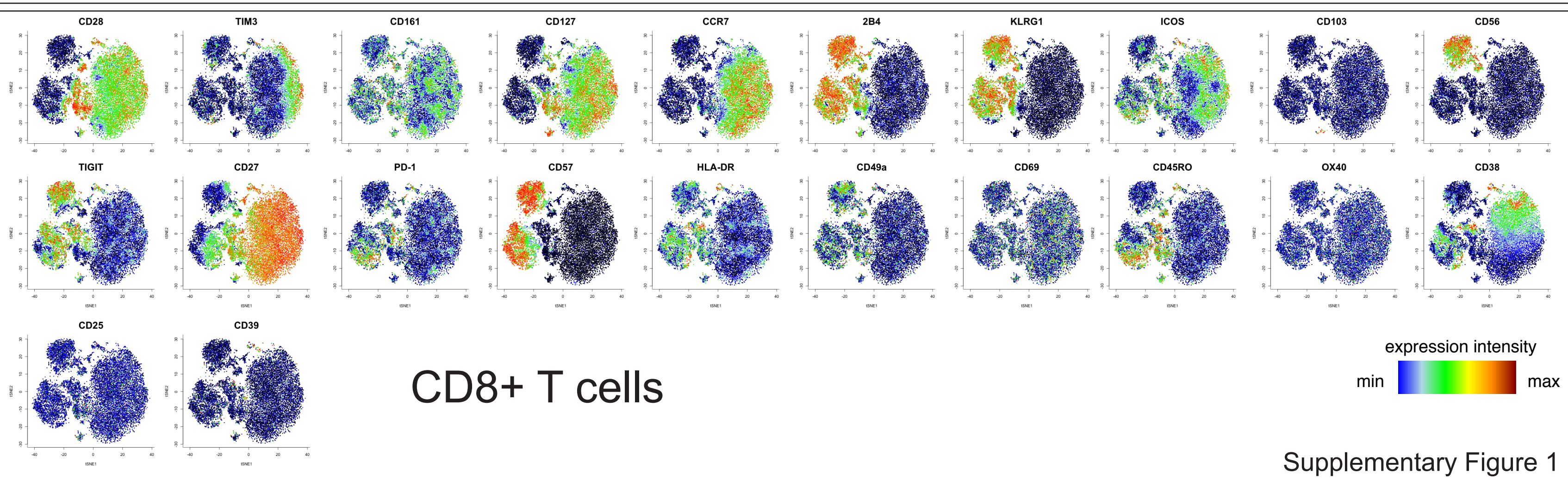

Supplement: Supplementary file 2 — Figure S2. High-dimensional immune profiles of CD8+ T cells from atezolizumab treated responders and non-responders. t-SNE maps display relative expression intensities of all phenotypic markers assessed. Shown are representative plots for CD8+ T cells and total CD45+ immune cells from one responder and one non-responder combined at baseline and on atezolizumab treatment. (PDF 25443 kb) [file 40425_2019_695_MOESM2_ESM.pdf]

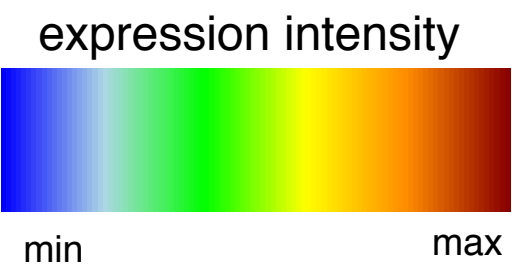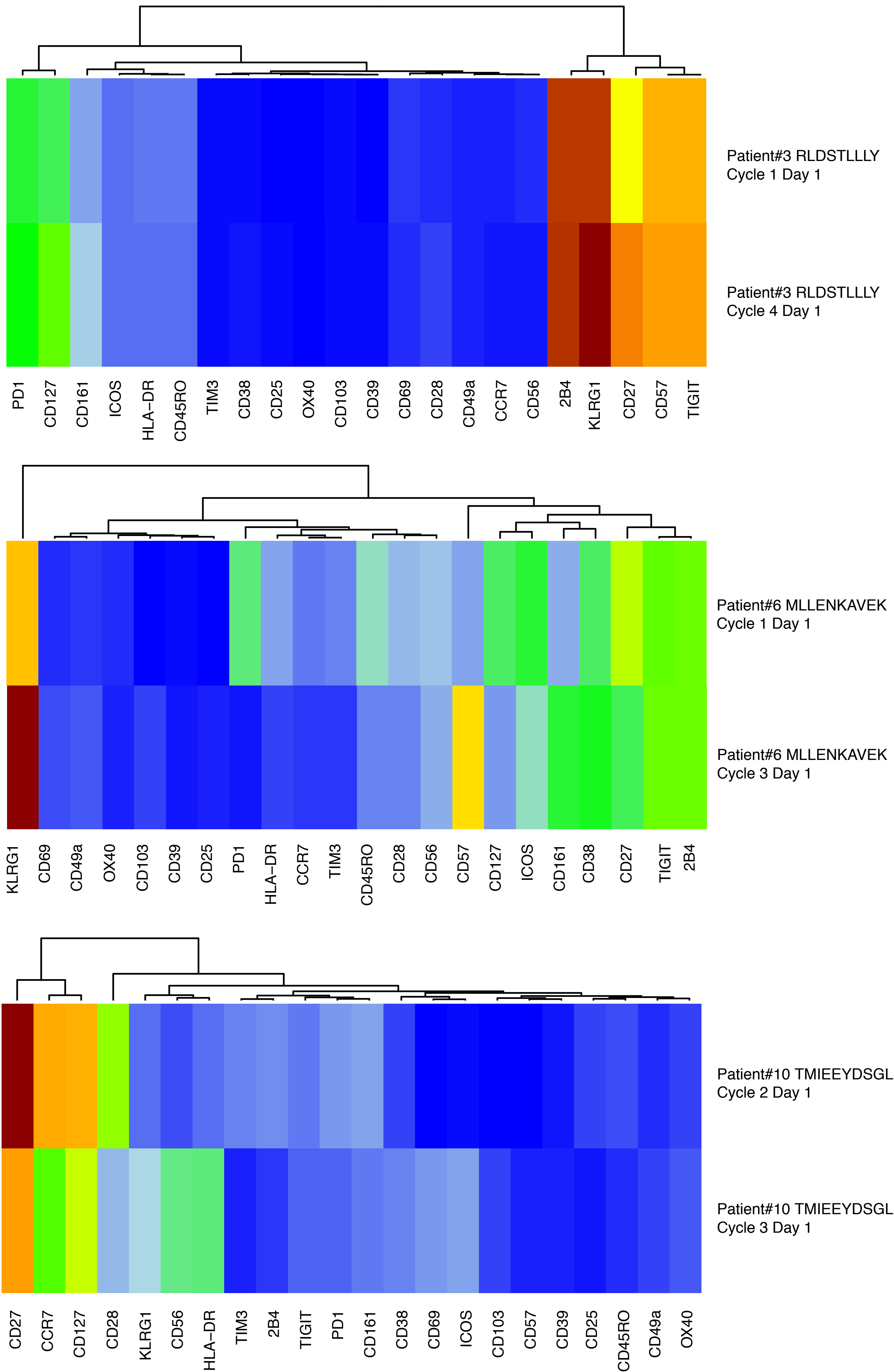

Supplementary Figure 2

Supplement: Supplementary file 3 — Figure S3. Phenotypic profiles of neoantigen-specific T cells in patients pre- and post atezolizumab treatment. Heatmaps show median expression intensities of all phenotypic markers probed in samples derived from the same patients. Markers are ordered based on unsupervised hierarchical clustering. (PDF 510 kb) [file 40425_2019_695_MOESM3_ESM.pdf]
